# Breast cancer mortality trends in Peruvian women

**DOI:** 10.1186/s12885-020-07671-x

**Published:** 2020-12-01

**Authors:** J. Smith Torres-Roman, Jose Fabian Martinez-Herrera, Greta Carioli, Jorge Ybaseta-Medina, Bryan Valcarcel, Joseph A. Pinto, Alfredo Aguilar, Katherine A. McGlynn, Carlo La Vecchia

**Affiliations:** 1 Universidad Cientifica del Sur, Lima, Peru; 2 Instituto de Investigación, Universidad Católica Los Ángeles de Chimbote, Chimbote, Peru; 3 Latin American Network for Cancer Research (LAN–CANCER), Lima, Peru; 4 Cancer Center, Medical Center American British Cowdray, Mexico City, Mexico; 5 Department of Clinical Sciences and Community Health, Università degli Studi di Milano, 20133 Milan, Italy; 6 Universidad Privada San Juan Bautista, Lima, Peru; 7 Unidad de Investigación Básica y Traslacional, Oncosalud-AUNA, Lima, Peru; 8 Division of Cancer Epidemiology and Genetics, National Cancer Institute, Bethesda, MD USA

**Keywords:** Breast cancer, Geographic spatial analysis, Mortality rate, Epidemiology, Female, Peru

## Abstract

**Background:**

Breast cancer (BC) is the most common malignancy in Latin American women, but with a wide variability with respect to their mortality. This study aims to estimate the mortality rates from BC in Peruvian women and to assess mortality trends over 15 years.

**Methods:**

We calculated BC age-standardized mortality rate (ASMR) per 100,000 women-years using the world standard SEGI population. We estimated joinpoint regression models for BC in Peru and its geographical areas. The spatial analysis was performed using the Moran’s I statistic.

**Results:**

In a 15-year period, Peru had a mortality rate of 9.97 per 100,000 women-years. The coastal region had the highest mortality rate (12.15 per 100,000 women-years), followed by the highlands region (4.71 per 100,000 women-years). In 2003, the highest ASMR for BC were in the provinces of Lima, Arequipa, and La Libertad (above 8.0 per 100,000 women-years), whereas in 2017, the highest ASMR were in Tumbes, Callao, and Moquegua (above 13.0 per women-years). The mortality trend for BC has been declining in the coastal region since 2005 (APC = − 1.35, *p* < 0.05), whereas the highlands region experienced an upward trend throughout the study period (APC = 4.26, *p* < 0.05). The rainforest region had a stable trend. Spatial analysis showed a Local Indicator of Spatial Association of 0.26 (p < 0.05).

**Conclusion:**

We found regional differences in the mortality trends over 15 years. Although the coastal region experienced a downward trend, the highlands had an upward mortality trend in the entire study period. It is necessary to implement tailored public health interventions to reduce BC mortality in Peru.

## Background

Breast cancer is the most common malignancy and the leading cause of cancer-related death among women worldwide [1]. In 2018, GLOBOCAN estimated 2.1 million breast cancer cases in women, accounting for almost 25% of all cancers [1]. Compared to high-income countries, breast cancer survival rates are lower in low- and middle-income countries (LMICs) [2], due to late diagnosis and inadequate access to cancer care [2, 3].

In the last years, breast cancer mortality has experienced downward trends in all Latin American countries (except Cuba); however, breast cancer continues to be one of the neoplasms with the highest mortality in Latin America [4–6]. Between 1980 and 2010, the highest mortality rates were in Argentina and Uruguay (around 20 deaths per 100,000 women-years), whereas the lowest rates were in Colombia, Mexico, and Ecuador (around 10 per 100,000 women-years) [4].

According to the GLOBOCAN 2018, breast cancer is the third cause of cancer death in Peruvian women, with an age-standardized mortality rate (ASMR) of 10.3 per 100,000 women [1], mainly affecting women in the coastal region [6]. However, there are no epidemiological studies on breast cancer mortality rates and trends from Peru and its geographical areas in the last years. This study aimed to estimate mortality rates from breast cancer among Peruvian women by geographical areas and to assess trends over time in Peru and its regions.

## Methods

### Design and study setting

We retrieved mortality data from the registry of deaths of the Peru Ministry of Health (MINSA, in Spanish) from the period 2003–2017: https://www.minsa.gob.pe/portada/transparencia/solicitud/. MINSA collects mortality data at the national level using different sources: 1) all health establishment records, 2) the Registro Nacional de Identificación y Estado Civil, and 3) the Public Ministry [7]. These data cover the number of deaths for each disease aggregated by gender into 5 age groups (0–11, 12–17, 18–29, 30–59, and ≥ 60) as in similar studies in Peru [7, 8]. We aggregated the 3 first age group in the young (0–29 years) group and calculated the age-adjusted mortality rates for the groups 0–29, 30–59, and ≥ 60. We used the code C50 to identify deaths from breast cancer, according to the International Classification of Diseases, 10th Revision [9]. Population denominators were obtained from census data in 2005, 2010, and 2015, conducted by the National Institute of Statistics and Informatics, which is the central and governing body of the National Statistical System, responsible he country’s official statistics [10].

**Fig. 1:**
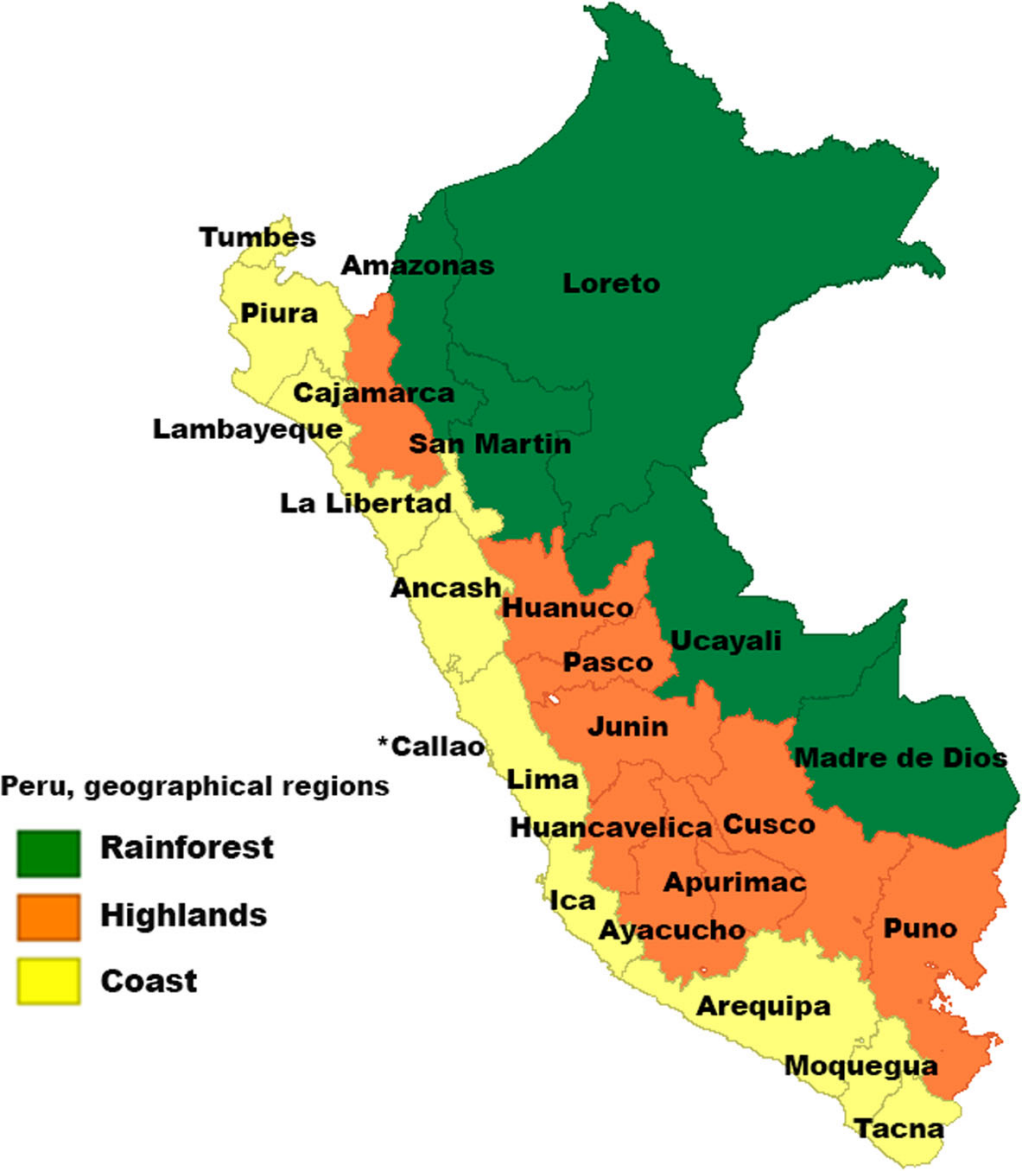
Peru geographical areas by provinces and regions. The asterisk denotes Callao province. Map created through GEODA version 1.14.0. Available in: https://geodacenter.github.io/index.html

Peru has 25 provinces located in three natural regions delimitated by the Andes mountains: coast, highlands, and rainforest [11]. The coast is the most densely populated region (56.3% of the total population) and represents the 11.7% of the territory [12]. The highlands, covering 27.9% of the national territory and 29.7% of the population [11], is a rural and urban high-altitude area throughout the Andes [12]. The rainforest, or the Peruvian Amazon, constitutes 60.3% of the territory, where 14.0% of the total population lives **(**Fig. 1**).**

### Ethical considerations

This manuscript is based on population databases and does not use any personal identifiable information. To obtain the raw data it was only necessary to fill out a form with the applicant’s data through:

https://www.minsa.gob.pe/portada/transparencia/solicitud/.

### Correction of under-reporting

Peru reported a rate of omission regarding the notification of the cause of death of 46%, ranging between 19% (province of Ica) and 78% (province of Loreto) [13]. The variable degree of coverage in the register of deaths from one province to another was corrected from 2003 to 2017 to be the known underreporting rate for each province, as a previous study [8]:

R = 100 – (OD/ED) × 100.

R = Underreporting rate.

OD = number of deaths observed for each province.

ED = number of deaths estimated for each province.

### Data analysis

With information on the number of deaths, corrected for the underreporting rate, we calculated age-standardized mortality rates per 100,000 women-years using the world standard SEGI population [14]. We analyzed mortality trends using the joinpoint regression program, version 4.7.0 [15, 16]. This model identified significant changes in trends, to identify the occurrence of possible joinpoints, allowing a minimum of zero joinpoints and a maximum of three joinpoints. For each time trend identified by the model, we estimated the annual percentage change (APC) [16]. We considered APCs statistically significant at a *p*-value < 0.05. For the provinces with more than 2 joinpoints, the average annual percentage changes (AAPC) were calculated. The significance levels utilized are based on the Monte Carlo permutation model and the calculation of the annual percentage change of ratio, utilizing the logarithm of the ratio [16, 17].

The GeoDA software was used to assess the spatial distribution [18]. The spatial analysis was performed using the Moran’s I statistic and the Local Indicator of Spatial Association (LISA), which assess whether mortality rates are clustered, dispersed, or random. The spatial representation was made using Moran’s local index known as LISA, this spatial typology consisted of five categories of health regions: (i) clusters with high breast cancer mortality rates surrounded by provinces with higher than average rates (‘high–high’); (ii) clusters with low breast cancer mortality rates surrounded by provinces with higher than average rates (‘low–high’); (iii) clusters with low breast cancer mortality rates surrounded by provinces with lower than average rates (‘low–low’) (positive autocorrelation); (iv) clusters with high breast cancer mortality rates surrounded by provinces with lower than average rates (‘high–low’)]; and (v) ‘not significant’. Values of Moran’s I range approximately from 1 (positive spatial autocorrelation, perfect grouping of similar rates) to 1 (negative spatial autocorrelation, spatial dispersion). We used a reference distribution using 999 random permutations to indicate statistical significance.

## Results

In the 2003–2017 period, we estimated 20,541 cumulative deaths from breast cancer in Peruvian women. The average mortality rate (per 100,000 women-years) over 15 years was of 9.97, in Peru. According to its regions, the coastal region had the highest rate (12.15), followed by the highlands (4.71), and the rainforest (7.94) regions. The provinces with the highest mortality rates in the overall period were Lima, Callao, and Tumbes, while the lowest rates were in Apurimac and Huancavelica (Table 1).

**Table 1:** Breast cancer deaths and mortality rates in Peru and its geographical areas from 2003 to 2017

Geographical areas	Deaths^**a**^		Mortality rates^**b**^	
	Uncorrected	Corrected	Uncorrected	Corrected
**Peru**	13,059	20,541	6.30	9.97
**Regions**				
**Coast**	11,466	16,905	8.21	12.15
**Highlands**	1229	2498	2.33	4.71
**Rainforest**	364	1138	2.48	7.94
**Provinces**				
**Amazonas**	66	253	2.80	10.91
**Ancash**	215	455	2.66	5.67
**Apurimac**	33	80	1.13	2.74
**Arequipa**	649	932	6.96	10.06
**Ayacucho**	56	139	1.39	3.45
**Cajamarca**	219	551	2.22	5.57
**Callao**	720	915	9.87	12.64
**Cusco**	173	372	2.01	4.27
**Huancavelica**	30	62	1.14	2.40
**Huanuco**	154	301	3.02	5.87
**Ica**	484	559	8.73	10.10
**Junin**	320	552	3.71	6.40
**La Libertad**	950	1365	7.57	10.99
**Lambayeque**	812	1030	8.97	11.46
**Lima**	6518	9896	9.08	13.83
**Loreto**	85	386	1.83	8.32
**Madre de Dios**	23	31	3.67	5.13
**Moquegua**	72	107	6.09	9.06
**Pasco**	49	104	2.77	5.93
**Piura**	840	1310	7.12	11.18
**Puno**	195	336	2.11	3.61
**San Martin**	119	326	2.75	7.56
**Tacna**	123	184	6.52	9.75
**Tumbes**	83	146	7.09	12.19
**Ucayali**	71	142	2.69	5.30

^a^Cumulative deaths for a 15-year period (2003–2017)

^b^Age-standardized rates per 100,000 women-years

**Fig. 2:**
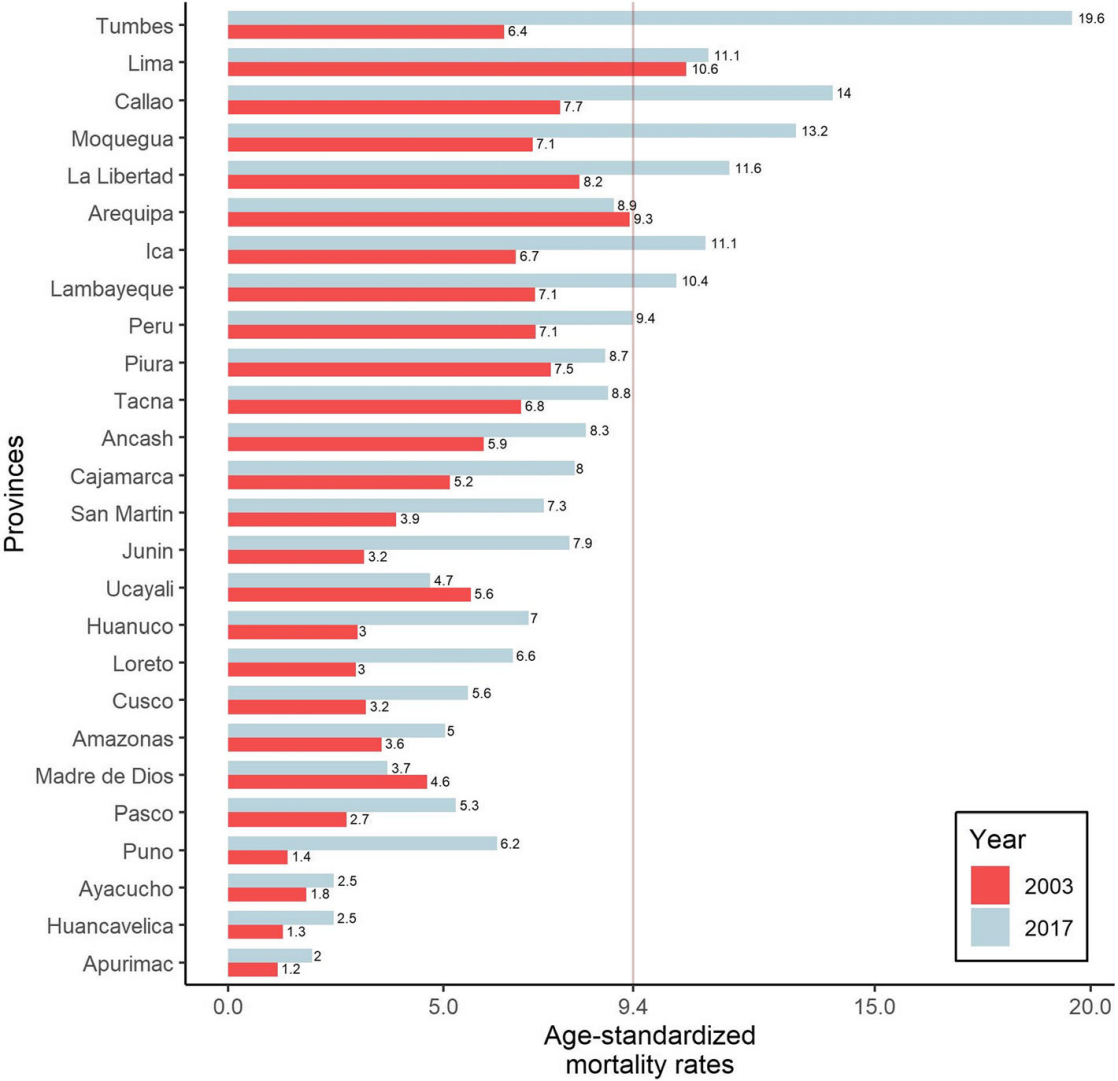
Age-standardised mortality rates (ASMRs) for breast cancer in 2003 and 2017

Figure 2 shows age-standardised mortality rates (ASMRs) for breast cancer from 2003 to 2017 in Peru and its 25 provinces. The ASMRs only declined in three provinces, Arequipa (from 9.3 to 8.9), Ucayali (from 5.6 to 4.7), and Madre de Dios (from 4.6 to 3.7). Seven coastal provinces (Tumbes, Lima, Callao, Moquegua, La Libertad, Ica, and Lambayeque) had higher mortality rates compared to the average rate of Peru in 2017 (dashed line in Fig. 2).

**Fig. 3:**
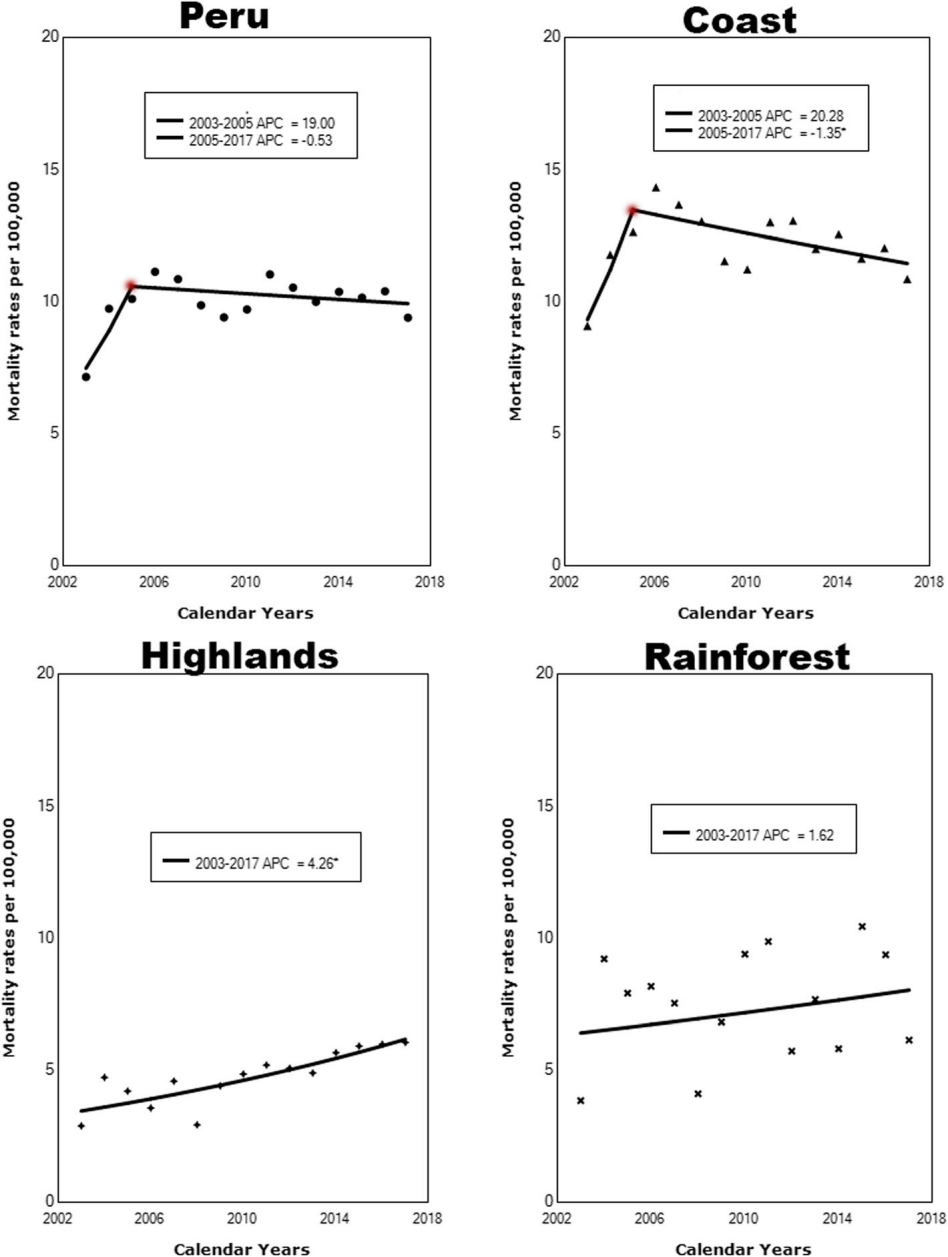
Joinpoint analysis for breast cancer in Peru and its regions between 2003 and 2017

Figure 3 shows the mortality trends for Peru and its regions between 2003 and 2017. The joinpoint analysis identified an initial upward trend in Peru (APC = 19) between 2003 and 2005, followed by a downward trend since 2005 until 2017 (APC = − 0.5). Similarly, the coastal region showed an annual increase of 20.2% from 2003 to 2005, and then start to significantly decrease until 2017 (ACP = − 1.35, *p* < 0.05). In contrast, both the highlands (APC = 4.26, p < 0.05) and rainforest (APC = 1.62) regions had a steady mortality increase over the study period.

Table 2 displays the age-standardized mortality rates for breast cancer per 100,000 women-years in the Peruvian provinces and its AAPC, between 2003 and 2017. The highest rise were in Puno (from 1.39 in 2003 to 6.24 in 2017, AAPC = 6.2, 95%CI: 3.0 to 9.6%), Tumbes (from 6.41 in 2003 to 19.58 in 2017, AAPC = 1.3, 95%CI: − 7.5 to 11.0%), and Junin (from 3.16 in 2003 to 7.92 in 2017, AAPC = 6.7, 95%CI: 3.4 to 10.2%). Moreover, Ancash and Apurimac provinces also showed upward mortality trends. Ancash had an increase from 5.93 to 8.31 (AAPC = + 4.1, 95% CI: 0.7, 7.5); Apurimac increased from 1.15 to 1.96 (AAPC = + 9.2, 95% CI: 1.6, 17.2).

**Table 2:** Age-standardized (world population) mortality rates per 100,000 women from breast cancer in Peruvian provinces, 2003 and 2017, corresponding percent changes (2017 vs 2003), and its Average Annual Percentage Changes

Provinces	2003	2017	%change 2003/2017	AAPC(95% CI)
**Amazonas**	3.56	5.04	41.51	2.4(−6.5, 12.1)
**Ancash**	5.93	8.31	39.99	4.1 (0.7, 7.5)
**Apurimac**	1.15	1.96	70.16	9.2 (1.6, 17.2)
**Arequipa**	9.32	8.95	−4.03	−0.8(−2.8, 1.2)
**Ayacucho**	1.82	2.46	35.11	2.9(−2.4, 8.4)
**Cajamarca**	5.15	8.04	55.99	3.3(−0.2, 6.8)
**Callao**	7.70	14.03	82.24	4.2(−2.2, 11.1)
**Cusco**	3.20	5.57	73.89	1.3(−3.1, 6.0)
**Huancavelica**	1.27	2.45	92.30	−1.3(−9.5, 7.7)
**Huanuco**	3.01	6.98	132.05	2.1(−3.2, 7.3)
**Ica**	6.68	11.07	65.83	2.7(−1.0, 6.4)
**Junin**	3.16	7.92	150.61	6.7 (3.4, 10.2)
**La Libertad**	8.15	11.63	42.71	1.3(−0.4, 3.1)
**Lambayeque**	7.12	10.40	46.13	−0.1(−3.1, 3.0)
**Lima**	10.62	11.14	4.86	0.7(−1.9, 3.4)
**Loreto**	2.97	6.61	122.46	2.5(−6.9, 12.8)
**Madre de Dios**	4.62	3.70	−19.81	0.3(−8.4, 9.8)
**Moquegua**	7.07	13.17	86.32	0.4 (−7.7, 9.2)
**Pasco**	2.75	5.29	92.46	2.3(−6.4, 11.9)
**Piura**	7.48	8.75	16.93	1.4(−0.8, 3.6)
**Puno**	1.39	6.24	348.99	6.2 (3.0, 9.6)
**San Martin**	3.91	7.32	87.43	3.3(−1.7, 8.6)
**Tacna**	6.80	8.81	29.55	−1.4(−5.4, 2.8)
**Tumbes**	6.41	19.58	205.32	1.3(−7.5, 11.0)
**Ucayali**	5.63	4.69	−16.65	−3.3(−7.8, 1.5)

*AAPC* Average Annual Percent Change

**Fig. 4:**
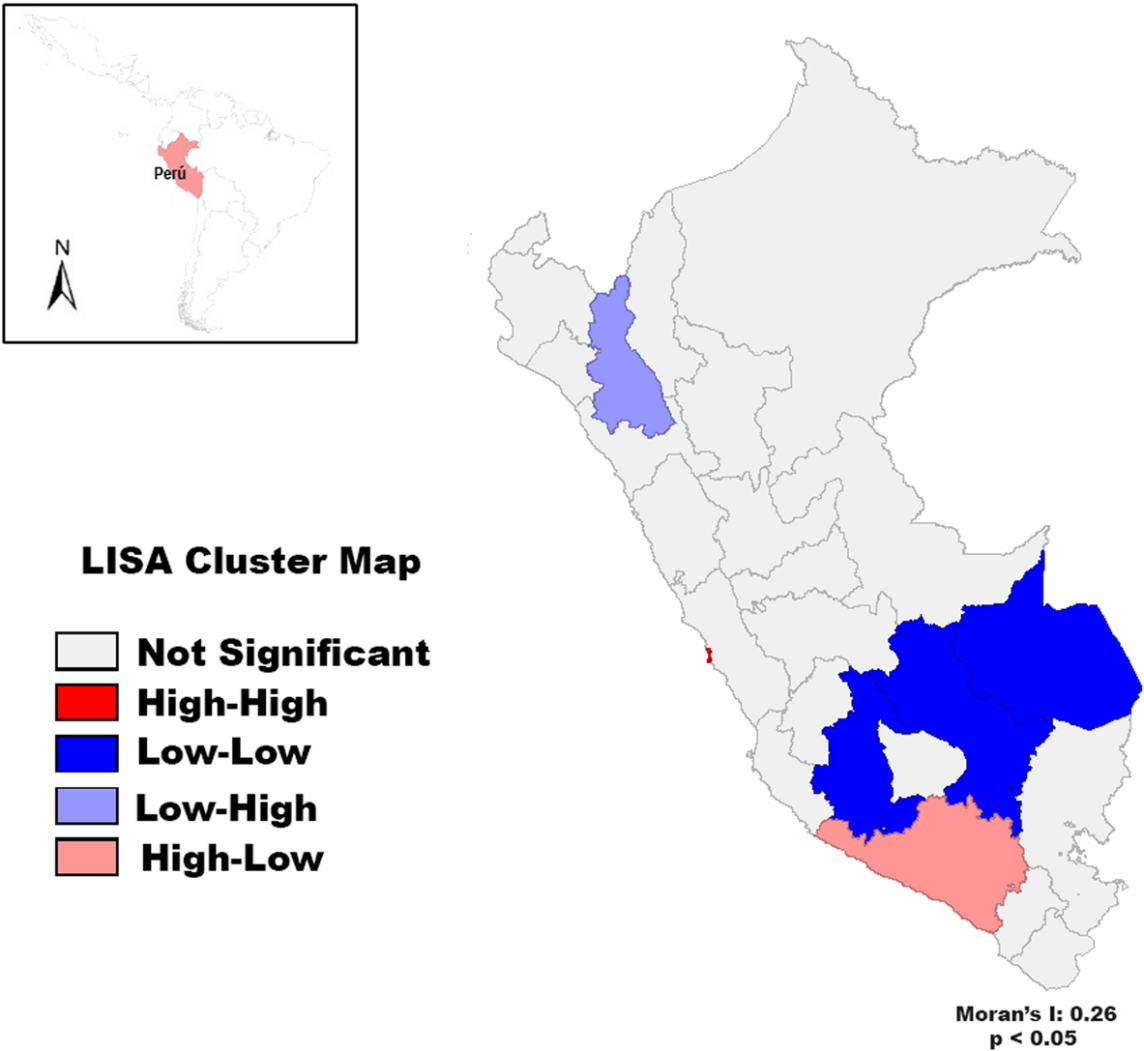
Cluster map for breast cancer in Peruvian women between 2003 and 2017. Map created through GEODA version 1.14.0. Available in: https://geodacenter.github.io/index.html

Figure 4 shows significant clustering and spatial outliers in the map according to the breast cancer mortality rates. A statistically significant cluster for “High-High” (**red**) occurrence was detected in Callao province, while “Low-Low” (**blue**) were identified in 3 provinces (Madre de Dios, Cusco, and Ayacucho), which had low rates, also surrounded by provinces with lower than average rates. Spatial analysis showed a LISA for provinces breast cancer mortality rates of 0.26 (*p* < 0.05).

## Discussion

We report the mortality rates of breast cancer over 15 years, with their trends using joinpoint regression models, and spatial analysis for Peru and its geographical areas. Our study showed stable trend in breast cancer mortality in Peru over the last two decades, but with differences across its regions. The coastal region showed the highest mortality rates in the analysed period, whereas the highlands region, despite having the lowest rates, experienced an upward trend.

The mortality rates increased during the first years of the study, probably due to an improvement in diagnosis and certification of deaths in various provinces of Peru [19]. In addition, in some provinces, the rates were so low that they likely represent underreporting of mortality. However, it is plausible that data quality has increased in last years [19].

Our findings differed from other areas of the world (most countries from Western Europe, North America, or some other Latin American countries) [5, 6, 20]. A study on breast cancer mortality trends and predictions for 2020 in the Americas and Australasia, including 7 Latin American countries [20], reported between 2000 and 2004, the highest mortality rates (per 100,000 women-years) in Argentina (19.75) and Uruguay (21.6), whereas Colombia (9.68) and Mexico (9.03) had the lowest mortality rates, with stables rates, and further expected 10% reduction in the overall rates for 2020. Peru is, therefore, among the countries with the lowest rates in the region.

Several factors influence the variations in breast cancer death certification across Latin American countries. These factors include a high burden of locally advanced/advanced breast cancer, inadequate access to healthcare resources, deficient access to specialized cancer care, and insufficient research [21]. Peru has a fragmented healthcare system with five major providers that have independent system structures and offer healthcare through different plans. Furthermore, approximately 25% of the population is uninsured [22] and lacks access to early diagnosis [23]. For instance, mammography has been reported more frequently among women with higher educational status, greater economic income, and living in major urban cities of the country with easier access to healthcare [24]. A persistent problem in Peru is the diagnosis at a late stage in one-third of breast cancer cases. Justo et al. reported that 32–33% of the patients had stage III at diagnosis, while 7–16% had stage IV [3]. These factors probably affect breast cancer case fatality, but without information on the incidence, it is difficult to assess the impact of these factors on mortality.

The coastal region had the highest mortality rates for breast cancer, principally in the Lima and Callao provinces, but over recent years showed downward trends. This region has the lowest poverty rates compared to the highlands or the rainforest [25] and the highest proportion of the oncological workforce (72% of clinical oncologists and 85% of radio-oncologists) [26]. Therefore, the population living on the coast has higher access to healthcare and better cancer detection, positively affecting mortality.

There are other possible explanations for the difference in mortality rates in the coastal region, such as racial differences [27], a higher percentage of obesity [28], higher alcohol consumption [29], and genetic susceptibility [30, 31]. In fact, the coastal region has the highest proportion of black women [32], mainly in the North and Centre. Also, several studies have reported a high prevalence of triple-negative breast cancer (TNBC) in Peruvian women [33, 34], with most cases identified in the coastal region [34]. Moreover, the women of the rainforest region were more likely to be diagnosed with TNBC compared to women of the highland region (31% vs. 14%), where the risk of mortality was 1.7 times higher in women of the rainforest [35]. It has been suggested that the distribution of breast cancer subtypes is due to the proportion of indigenous women in the two regions of Peru [35].

The Peruvian government made serious efforts to improve early detection, diagnosis, and treatment for cancer in the early 2010s. The Plan Esperanza aimed to decentralise healthcare to all the provinces, improve cancer screening, and provide treatment at a lower cost [36]. This practice tends to increase the diagnosis of cancer, because of better detection, which consequently increases the mortality rates. Hence, the higher mortality rate in the period 2013–2017 may reflect the effect of the Plan Esperanza, especially in the highland provinces. However, it is still too soon to observe a benefit from this nationwide program on mortality reduction. Other efforts have begun to establish an effective model in low resource areas, such as training health promoters for community outreach, training professionals to perform fine-needle aspiration biopsy sampling, and ensuring that patients adhere to treatment regimens [37]. These strategies will become part of the national strategy to reduce mortality from breast cancer since optimal access to care and early diagnosis and treatment are crucial for mortality control [38]. Besides, a cost-effectiveness analysis of breast cancer control interventions in Peru [39] showed that the current program could be improved by implementing triennial or biennial screening strategies. Such a strategy would offer critical treatment to cases detected in the early stages and, consequently, to offer higher quality health coverage to the population.

### Limitations and strengths

This study has several limitations: 1) the lack of a high-quality national death registration. However, the calculation was made with correction for underreporting, informed by the Ministry of Health. 2) The absence of a National Cancer Registry that provides nationwide incidence data. Interpreting mortality without incidence data in each province, could lead to a bias of interpretation of results. The Lima Metropolitan Cancer Registry (collecting data from Lima, capital from Peru) and the Arequipa Population Cancer Registry are the only cancer registry active in Peru, although have not reported data in quite a long time. 3) Although the SEGI world standard population was used, it is not really comparable to the SEGI standardized mortality rates from other countries, as the normal SEGI population used 18 age-groups, versus only 3 broad age groups (0–29, 30–59, and ≥ 60) in this study. These very broad age groups can still hide substantial ageing of the population and therefore are not really comparable to rates of other countries. As a strength of our work, this is the first large scale study for Peru and its geographical areas that evaluated the changes in breast cancer mortality.

## Conclusion

Our study showed a stable mortality trend of breast cancer in Peruvian women. Moreover, their rates still have lower compared to other Latin American countries. The highest mortality rates from breast cancer were observed in the coastal region, but a recent downward trend was observed since 2005. Also, increasing mortality trends were seen in the highland region. The disparities may be related to variable death certification validity, the access to early diagnosis, and treatment for breast cancer. Tailored public health interventions to reduce breast cancer mortality should be implemented in Peru.

## Data Availability

The datasets used and/or analysed during the current study are available from the corresponding author on reasonable request. You can also request the raw data through the following form: https://www.minsa.gob.pe/portada/transparencia/solicitud/, placing your personal data and the information to be requested.

## Abbreviations

ASMR: Age-standardized mortality rate
ASMRs: Age-standardized mortality rates
BC: Breast cancer
LMICs: Low- and middle-income countries
NSI: National Statistics Institute
TNBC: Triple-negative breast cancer
